# Lessons learned from detecting and responding to recurrent measles outbreak in Liberia post Ebola-Epidemic 2016-2017

**DOI:** 10.11604/pamj.supp.2019.33.2.17172

**Published:** 2019-05-29

**Authors:** Thomas Nagbe, George Sie Williams, Julius Monday Rude, Sumor Flomo, Trokon Yeabah, Mosoka Fallah, Laura Skrip, Chukwuemeka Agbo, Nuha Mahmoud, Joseph Chukwudi Okeibunor, Kwuakuan Yealue, Ambrose Talisuna, Ali Ahmed Yahaya, Soatiana Rajatonirina, Adolphus Clarke, Esther Hamblion, Tolbert Nyenswah, Bernice Dahn, Alex Gasasira, Ibrahima Socé Fall

**Affiliations:** 1 National Public Health Institute, Monrovia, Liberia; 2 World Health Organization, Monrovia, Liberia; 3 World Health Organization, Regional Office for Africa, Brazzaville, Congo; 4 Ministry of Health, Monrovia, Liberia

**Keywords:** Integrated disease surveillance and response, measles, alert and epidemic thresholds, immunization, outbreak

## Abstract

**Introduction:**

Measles is an acute viral disease that remains endemic in much of sub-Sahara Africa, including Liberia. The 2014 Ebola epidemic disrupted an already fragile health system contributing to low uptake of immunization services, population immunity remained low thus facilitating recurrent outbreaks of measles in Liberia. We describe lessons learnt from detecting and responding to recurrent outbreaks of measles two years post the 2014 Ebola epidemic in Liberia.

**Methods:**

We conducted a descriptive study using the findings from Integrated Diseases Surveillance and Response (IDSR) 15 counties, National Public Health Institute of Liberia (NPHIL), National Public Health Reference Laboratory (NPHRL) and District Health Information Software (DIHS2) data conducted from October to December, 2017. We perused the outbreaks line lists and other key documents submitted by the counties to the national level from January 2016 to December 2017.

**Results:**

From January 2016 to December 2017, 2,954 suspected cases of measles were reported through IDSR. Four hundred sixty-seven (467) were laboratory confirmed (IgM-positive), 776 epidemiologically linked, 574 clinically confirmed, and 1,137 discarded (IgM-negative). Nine deaths out of 1817 cases were reported, a case fatality rate of 0.5%; 49% were children below the age of 5 years. Twenty-two percent (405/1817) of the confirmed cases were vaccinated while the vaccination status of 55% (994/1817) was unknown.

**Conclusion:**

Revitalization of IDSR contributed to increased detection and reporting of suspected cases of measles thus facilitating early identification and response to outbreaks. Priority needs to be given to increasing the uptake of routine immunization services, introducing a second dose of measles vaccine in the routine immunization program and conducting a high-quality supplementary measles immunization campaign for age group 1 to 10 years to provide protection for a huge cohort of susceptible.

## Introduction

Measles is an acute viral infectious disease caused by a virus in the paramyxovirus family and an important cause of childhood morbidity and mortality [1]. In 2015, there were 206,360 cases of measles reported globally with 42,083 reported in the African region [2]. Measles is part of the list of 14 epidemic prone diseases for weekly reporting under IDSR in Liberia [3]. In September 1998, the World Health Organization-Regional Committee for Africa (WHO/AFRO) adopted the Integrated Disease Surveillance and Response (IDSR) strategy for priority diseases, conditions and events as one of the key aspects of disease control in the WHO African Region that leads to early detection, appropriate investigation, laboratory confirmation and timely response to public health conditions and events [4-6]. The Liberia Ministry of Health (MOH) adapted the second edition of the generic integrated disease surveillance and response technical guideline in 2014 supported by World Health Organization-Regional Office for Africa (AFRO) in collaboration with the United States Centers for Disease Control and Prevention (CDC) in Atlanta. The guidelines aimed at contributing to reduction of mortality, morbidity and disability from diseases, public health events and conditions through timely, accurate, complete reporting and analysis of data for public health action. The guidelines serve as a general reference for surveillance activities across all levels of the health system [4] and were revised in 2016 to suit the prevailing national situation. As a lesson learnt from EVD outbreak in Liberia [7], the strengthened IDSR has led to increased reporting of suspected cases, laboratory confirmation as well as linkage of epidemiological and laboratory data for detection and response to measles outbreaks throughout the country. This paper aims at highlighting the lessons learned from enhanced surveillance and response to measles through IDSR. These lessons and recommendations will contribute to the quality of measles surveillance and response as well as the global measles elimination efforts.

## Methods

**Study setting:** Liberia has 15 political divisions or counties which are further subdivided into 91 health districts. The projected estimated population of Liberia in 2017 according to Liberia’s Health Management Information System (HMIS) was 4,514,112. In 2017, there were a total of 761 health facilities, all of which submitted weekly surveillance reports on 14 nationally identified immediately reportable diseases, conditions and events through the IDSR strategy. There is also a National Public Health Reference Laboratory (NPHRL) responsible for the confirmation of suspected cases requiring testing. The NPHRL currently has testing capacity for eight priority diseases (measles, Ebola, yellow fever, lassa fever, Rubella, Shigellosis, cholera and meningitis).

**Study design:** we conducted a descriptive and cross-sectional study to analyze the data from all the measles outbreaks reported from January 2016 to December 2017 and immunization data from 2012 to 2017.

**Standard case definition:** we adopted the standard case definitions from the National Technical Guidelines for IDSR and WHO’s recommended standards for surveillance of selected vaccine-preventable diseases: **suspected case:** any person with fever and generalized maculo-papular (non-vesicular) rash plus one of the following: Cough coryza or conjunctivitis (red eyes); or any person in whom a clinician suspects measles. **Confirmed case:** a suspected case with laboratory confirmation (positive IgM antibody) or epidemiological link to confirmed cases in an outbreak. **Epidemiologically confirmed case:** any case that meets the suspect case definition and is linked to a laboratory confirmed case. **Clinically confirmed case:** a case that meets the suspect case definition and a clinician is convinced to be measles in absence of laboratory confirmation [3].

**Thresholds:** an IDSR alert threshold was defined as one suspected measles case reported by a district in a week. The epidemic threshold was defined as five or more suspected cases or three confirmed cases reported from a district in one month [8].

**Response to measles outbreaks:** the IDSR alert and epidemic thresholds along with spatial and temporal clustering of cases were used to detect outbreaks. For each outbreak detected, a response from the district rapid response team (DRRT) was mounted within 48 hours after the confirmation of the outbreak and in some cases supplemented by the county response team with in 72 hours. Response to measles outbreaks included active case search in communities and line listing of all cases, record review at health facility to identify missed cases, and symptomatic management of cases including administration of high dose of vitamin A. Reactive vaccination campaigns were conducted in selected communities in some districts with variation among targeted age group for each campaign depending on the population most affected and most vulnerable. Engagement of communities using local health volunteers to report suspected cases, seek care and utilize existing immunization services became a mainstay of the response strategy to outbreaks.

**Data collection:** we obtained weekly measles IDSR surveillance data reported by the county surveillance officers covering each of the 15 political divisions of the country. These reports were also complemented by weekly laboratory results released by the National Public Health Reference Laboratory. When a health district crossed the epidemic threshold and reported outbreaks, the measles outbreak reports and line list were obtained and used as a data source.

**Immunization:** immunization records were obtained from the Health Management Information System (HMIS). We reviewed immunization records from 2012 to 2017 across the country. Records of reactive immunization during outbreaks were also obtained.

**Data analysis:** univariate and multivariate analysis were performed using Microsoft™ Excel 2013 and Epi Info™ 7.0. We analyzed the data to describe demographic and epidemiological characteristics such as attack rate, age group, sex, and vaccination status. Patients confirmed cases were divided into 4 age groups: < 1 year, 1-4 year, 5-9 year, ≥ 10 year. We performed F-test to compare the distribution of cases by age range, gender and vaccination status, and student-t test to compare the variation of cases from 2016 to 2017. Positive predictive value which was defined as the proportion of measles case-patients who had a positive measles serological result (IgM +) [9] was also calculated for each county and presented in tables. Arc GIS was used to provide spatial analysis and presentation of the data on maps. Following discussions with ministry of health ethics committee and National Public Health Institute of Liberia for which permission to proceed with the research and data analysis was granted the IRB review request was not considered.

## Results

**Figure 1:**
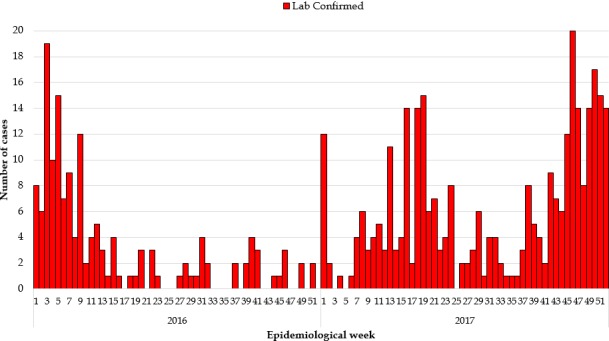
Laboratory confirmed measles cases 2016 and 2017

**Figure 2:**
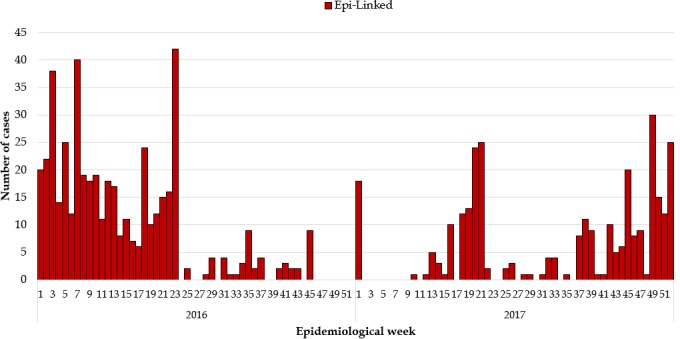
Measles Epi-Linked cases 2016 and 2017

**Figure 3:**
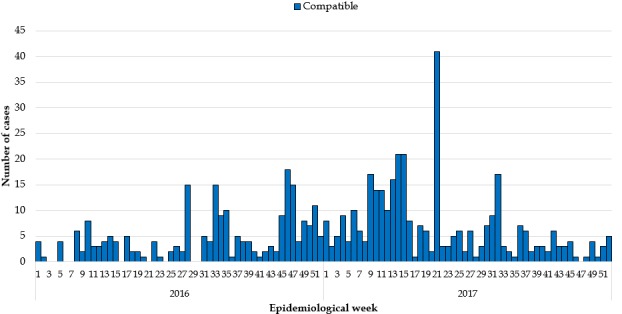
Clinically compatible measles cases 2016 and 2017

**Figure 4:**
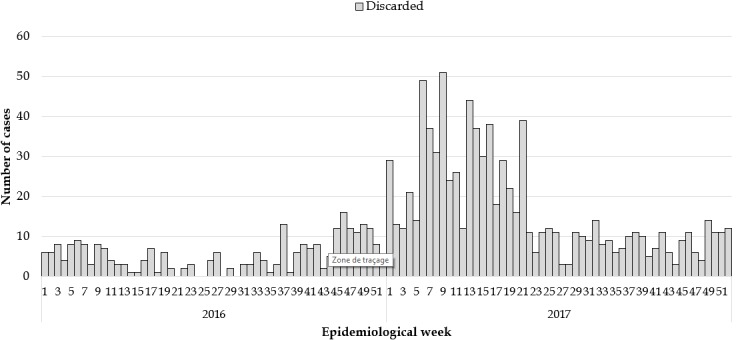
Discarded measles cases 2016 and 2017

**Figure 5:**
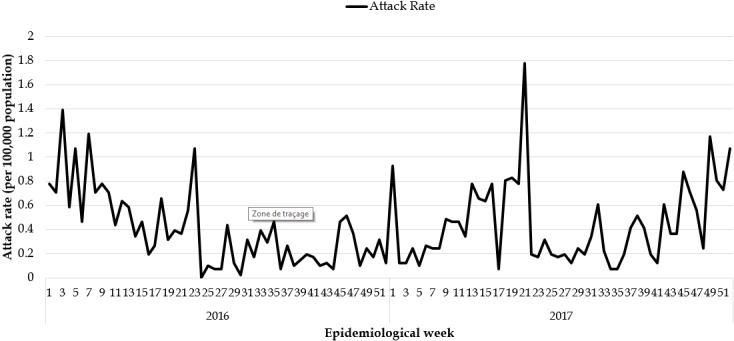
Measles attack rate in Liberia 2016 and 2017

**Figure 6:**
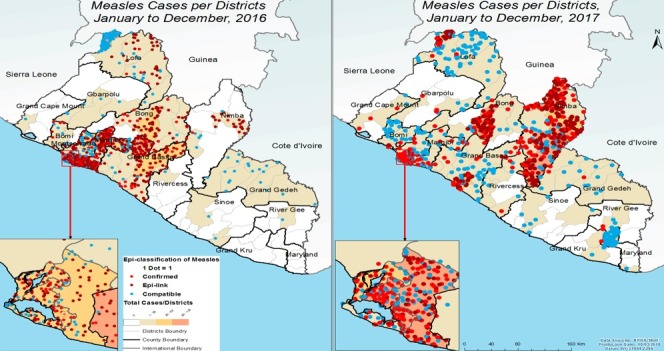
Comparison of geographical distribution of confirmed cases of measles in Liberia, 2016 and 2017

**Epidemiological description of the measles outbreaks:** a total of 2,954 suspected cases of measles were reported from all 15 counties. Using WHO classification scheme for measles [6], 467 were laboratory confirmed (IgM-positive), 776 epidemiologically confirmed, 574 clinically confirmed, and 1,137 discarded (IgM-negative) (Figure 1, Figure 2, Figure 3, Figure 4, Figure 5). The ratio of males to females was not statistically significant among confirmed cases (p = 0.25). The median age among confirmed cases was 5 years with an inter-quartile range of 1-8. Among the confirmed cases, the most affected age group was 1-4 year with 37% of cases (667/1817) (p < 0.001). Vaccination status among confirmed cases were as follow: vaccinated-405 (22%) (p < 0.001), not-vaccinated-418 (23%) (p < 0.001), and unknown-994 (55%) (p = 0.85) (Table 1). Nine deaths were reported among confirmed cases in both reporting years with a case fatality rate of 0.5% (9/1817), six of them not vaccinated and three with unknown vaccination status. Thirteen health districts in six counties reported outbreaks in 2016 and in 2017. In 2017, 29 outbreaks were recorded involving 955 confirmed measles cases. Case fatality rate among confirmed cases was 0.42% (4/955). Most outbreaks 83% (24/29) were responded to within 48 hours of detection (Table 2). Although the counties with outbreaks in 2016 and 2017 were the same, there was variation in the number of cases and distribution among the health districts with outbreaks between the two years (Figure 6), with more districts involved and cases reported in 2017 compared to 2016. From 2016 to 2017, the number of confirmed cases increased in all the counties, except Grand Bassa, Grand Gedeh, Lofa and Margibi. There was an overall not significant increase in number of cases from 2016 to 2017 (p = 0.39) in the entire country.

**Table 1:** Outbreak log of measles, Liberia, 2016 – 2017

				Sex		Age	Vaccination Status					
	County	Date Reported (Date/Month/Year)	Districts affected	Male (%)	Female (%)	Median Age	Vaccinated (%)	Not Vaccinated (%)	Unknown (%)	No. of deaths	CFR (%)	Duration from Notification to Response (days)
1	Bomi	9/1/2016	Senjeh, Klay	19 (54%)	16 (46%)	6	11 (31%)	3 (9%)	21 (60%)	0	0	2
2	Bong	17/6/2016; 11/5/2017; 14/9/2017; 15/11/2017	Zota, Suakoko	70 (47%)	78 (53%)	5	11 (7%)	39 (26%)	98 (66%)	0	0	1
3	Gbarpolu	3/1/2017	Bokomu	8 (53%)	7 (47%)	6	8 (53%)	0 (0%)	7 (47%)	0	0	2
4	Grand Bassa	10/1/ 2016; 10/5/ 2016; 4/3/2017; 4/3/2017; 12/6/2017	Buchanan, Wood camp, District number 3 and 4	114 (53%)	103 (47%)	5	9 (4%)	112 (52%)	96 (44%)	2	0.92	9
5	GCM	14/1/2017	Tewor	4 (67%)	2 (33%)	6	1 (17%)	0 (0%)	5 (83%)	0	0	
6	Grand Gedeh	11/1/2017	Tchien, Konobo	20 (51%)	19 (49%)	5	6 (15%)	5 (13%)	28 (72%)	0	0	
7	Grand Kru	16/2/2017	Buah	1 (33%)	2 (67%)	6	0 (0%)	1 (33%)	2 (67%)	0	0	
8	Lofa	16/1/2016; 31/3/2017;	Voinjama,Zorzor, Foya	103 (50%)	102 (50%)	5	71 (35%)	20 (10%)	114 (56%)	0	0	2
9	Margibi	5/1/2016, 3/10/2016; 8/5/2017	Gibi,Kakata, Firestone	130 (48%)	143 (52%)	5	37 (14%)	96 (35%)	140 (51%)	2	0.73	8
10	Maryland	3/3/2017	Pleebo	26 (55%)	21 (45%)	6	2 (4%)	0 (0%)	45 (96%)	0	0	2
12	Montserrado	5/1/2016; 18/2/2017; 6/3/2017; 25/10/2017; 1/11/2017; 24/11/2017; 1/12/2017;	Bushrod,St Paul, Common wealth, Somalia drive	255 (50%)	251 (50%)	5	195 (39%)	99 (20%)	212 (42%)	1	0.2	7
12	Nimba	17/2/2016; 24/4/2017; 21/7/2017; 11/8/2017; 4/11/2017; 15/10/2017	Tappita, Saniqueille Mah,Yarwin, Mensonnoh	145 (49%)	153 (51%)	5	52 (17%)	42 (14%)	204 (68%)	4	1.34	7
13	River Gee	13/3/2017	Webbo	3 (60%)	2 (40%)	6	2 (40%)	0 (0%)	3 (60%)	0	0	2
14	Rivercess	4/4/2017	Morweh	3 (27%)	8 (73%)	5	0 (0%)	0 (0%)	11 (100%)	0	0	2
15	Sinoe	19/5/2017	Greenville	4 (44%)	5 (56%)	6	0 (0%)	1 (11%)	8 (89%)	0	0	2
	Grand Total			904 (50%)	911 (50%)	5	405 (22%)	418 (23%)	994 (55%)	9	0.49	
												≤ 48 hours
												3 - 7 days
												>7 days

**Table 2:** Measles surveillance by county, Liberia, January 2016-December 2017

County	2016				2017			
	Total cases requiring sample collection	Total samples collected	% of samples collected	Ig M positve	Total cases requiring sample collection	Total samples collected	% of samples collected	Ig M positve
Bomi	29	26	90%	3	93	69	74%	1
Bong	34	31	91%	11	91	86	95%	27
Gbarpolu	10	5	50%	0	19	15	79%	3
Grand Bassa	47	45	96%	24	82	54	66%	19
Grand Cape Mount	5	4	80%	0	37	36	97%	3
Grand Gedeh	30	10	33%	0	74	62	84%	4
Grand Kru	3	3	100%	0	23	21	91%	1
Lofa	131	50	38%	4	196	134	68%	8
Margibi	132	114	86%	56	153	141	92%	15
Mary Land	5	5	100%	0	98	52	53%	1
Montserrado	196	132	67%	49	375	293	78%	148
Nimba	17	12	71%	3	192	172	90%	83
River Gee	2	2	10%	0	13	10	77%	1
River Cess	3	3	10%	0	39	33	85%	3
Sinoe	7	6	86%	0	45	40	89%	0
**Grand Total**	**648**	**451**	**70%**	**150**	**1530**	**1218**	**80%**	**317**

**Laboratory description:** blood specimens for measles testing were obtained from a total of 1,669 suspected cases (451 in 2016 and 1,218 in 2017) and sent to the National Public Health Reference Laboratory. Excluding epi-linked cases, the proportion of suspected cases with blood specimens collected increased from 70% in 2016 to 80% in 2017. The total number of measles specific IgM positive samples increased from 150 in 2016 to 317 in 2017. The positive predictive value among samples tested decreased from 33% in 2016 to 26% in 2017 (Table 3).

**Table 3:** Administrative coverage of measles-containing vaccine from 2013 to 2017 and reactive campaign vaccination coverage from 2016 to 2017

	MCV1 administrative Coverage (%)					Reactive local vaccination				
County	2013	2014	2015	2016	2017	No. of districts with campaign	No. of communities (2016)	No. of communities (2017)	Coverage (%) 2016	Coverage (%) 2017
**Bomi**	87%	70%	72%	93%	92.6%	-	-	-	-	-
**Bong**	92%	83%	108%	111%	113.3%	2	14	3	81%	113%
**Gbarpolu**	71%	58%	56%	92%	89.4%	-	-	-	-	-
**Grand Bassa**	64%	60%	75%	79%	82.2%					
**Grand Cape Mount**	82%	46%	58%	64%	62.0%	-	-	-	-	-
**Grand Gedeh**	71%	60%	51%	61%	65.6%	-	-	-	-	-
**Grand Kru**	80%	77%	58%	96%	106.6%	-	-	-	-	-
**Lofa**	70%	60%	63%	93%	91.5%	1	10	-	89%	-
**Margibi**	63%	55%	63%	98%	77.8%	3	37	1	78%	85%
**Maryland**	58%	57%	45%	65%	61.5%	-				
**Montserrado**	72%	47%	55%	74%	86.2%	0	0	0	0	0
**Nimba**	73%	58%	55%	73%	98.3%	4	2	14	31%	78%
**River Gee**	69%	40%	31%	42%	62.2%	-	-	-	-	-
**Rivercess**	75%	75%	82%	79%	74.9%	-	-	-	-	-
**Sinoe**	69%	73%	81%	77%	78.0%	-	-	-	-	-
Liberia	74%	61%	64%	80%	87.1%					
**% of district with MCV1 ≥80%**	**4**	**1**	**3**	**6**	**9**	-	-	-	-	-
**% of district with MCV1 <50%**	**0**	**3**	**2**	**1**	**0**	-	-	-	-	-

**Measles immunization coverage:** records from the HMIS showed that routine measles vaccination (MCV1) coverage declined from 74% in 2013 to 61% and 64% in 2014 and 2015 respectively before picking up at 80% in 2016 and 87% as of December 2017. Analysis of measles coverage by county shows variation in performance. The proportion of districts attaining routine measles vaccination coverage above 80% decrease from 4 (4.4%) in 2013 to 1 (1.1%) in 2014 and increased to three (3.3%) in 2015, 6 (6.7%) in 2016 and 9 (10%) in 2017. The proportion of districts reporting less than 50% measles vaccination coverage increase from 0 in 2013 to 3 in 2014 and then declined to 2, 1, and 0 in 2015, 2016, and 2017 respectively (Table 4). Due to recurrent measles outbreaks, 15 reactive vaccinations campaigns were conducted in five counties, targeting 63 affected and surrounding communities in 2016 and 18 communities in 2017. Seven counties out of the total 15 counties 7/15 (46.7%) did not reach the national measles coverage target of 80% in 2017. The situation was worse in 2013 where only 4/15 (27%) counties achieved this target.

**Table 4:** Reactive measles vaccinations during measles outbreaks, Liberia, 2016 – 2017

Year	Dates	County	Health District	Number of cases	Age group targeted	Number targeted	Number Vaccinated	Coverage (%)
2016	4-11 March	Bong	Suokoko	6	6-59 months	340	223	66
2016	7-11 March	Nimba	Zoe Geh	8	6-59 months	4257	1010	24
2016	14-18 March	Margibi	Mamba Kaba, Fire stone & Kakata	112	9-23 months	9916	7152	72
2016	22-23 March	Bong	Sanoyea	4	6-59 months	1128	973	86
2016	12-16 September	Lofa	Foya	30	6-59 months	12938	11540	86
2016	19-23 September	Nimba	Saclepea Mah	434	6-59 months	351	434	124
2016	3-7 October	Margibi	Firestone	4	9-11 months	2620	2620	100
2017	1-5 May	Nimba	Tappita	79	6-59 months	1290	1746	135
2017	8- 12 May	Margibi	Gibi	5	9-11 months	420	355	85
2017	14-18 August	Nimba	Zoe Geh	10	6-59 months	194	111	57
2017	18-28 September	Bong	Suokoko	4	6 months - 10 years	1002	839	84
2017	16-30 October	Bong	Suokoko	3	7 months - 10 years	1491	1969	123
2017	23-27 October	Nimba	Zoe Geh	11	6-59 months	400	300	75
2017	6-24 November	Bong	Zota	3	7 months - 10 years	1015	839	83
2017	11-15 December	Nimba	Sanniquellie	46	6-59 months	1816	715	39

## Discussion

Since the revitalization of IDSR in Liberia towards the end of 2015, detection, reporting, and response to outbreaks of measles in the following two years has improved. The total number of suspected measles cases reported in Liberia increased from 1,134 cases in 2016 to 1,820 cases in 2017. The improved detection and reporting of cases may be attributed to training of health workers across the country in IDSR, mentorship of frontline health workers on measles case definition and treatment as well as the introduction of community events based surveillance system where measles is one of the 14 triggers (using pictorial and simplified definitions) notifiable by community health workers to the nearest health facility. The age group 1-4 years old was the most affected constituting 37% of the confirmed cases followed by 5-9 years age group constituting 29% of the cases. Although, the national Expanded Program on Immunization (EPI) offers a single dose of measles-containing vaccine to children at age 9 months [10], the most affected age group were above those covered in routine immunization. The huge burden of cases above the target age for routine vaccination can be explained in terms of the number of missed children who have formed part of a large susceptible population. Additionally, failure to seroconvert may account for cases among those previously vaccinated despites having received a dose of the vaccine. According to a study conducted by Işik et al., among 115 children seroconversion rate was 77.6% after the first dose at 9 months of age and 81.9% after the second dose of measles vaccine at 15 months [11]. Studies indicate that more than 99% of persons who receive two doses of measles vaccine (with the first dose administered no earlier than the first birthday) develop serologic evidence of measles immunity [12]. However even when vaccination coverage is high measles outbreaks may still occur if the quality of the vaccination program (vaccine potency, route of administration, and dosage policy) does not offer enough protection among the population.

**Laboratory confirmation:** measles diagnosis is confirmed by Laboratory and in Liberia only the National Reference Laboratory (NRL) offer testing for measles in the country. Whenever measles is suspected, whole from the first 5-10 suspected measles cases is sent to the NRL for confirmation. When 3 measles cases are confirmed from the same district within a period of 30 days, a measles outbreak is declared and additional measles cases reported are epidemiologically liked and line listed [13]. In 2016, the national percentage of 70% fell below the WHO target of 80% for minimum proportion of suspected cases requiring samples collection [14], however, the target of 80% was achieved in 2017 highlighting improvement in surveillance and laboratory. Sensitivity of the surveillance system also increases with high number of suspected cases reported in 2017 compared to 2016. Positive predictive value in both years remains above the WHO AFRO Regional target of 10%, suggesting the endemicity of measles in the country. Virus detection, sequence information and oropharyngeal swab specimens are not currently in Liberia. This sequence information can be of much value to the national control programs, determine transmission pathways, and define geographical distribution of measles virus genotypes.

**Clinical management of measles cases:** the WHO recommendations for administration of vitamin A and supportive care to patients diagnosed of measles was used which is attributed to high recovery rate, low case fatality rate and post measles complications [15]. The supportive treatment provided for all measles cases at secondary and tertiary health facilities include the administration of additional fluids (such as oral rehydration solution) and antipyretics. Antibiotics were used for measles complicated by otitis media, pneumonia or other suspected sepsis and nutritional therapy given to children with malnutrition. All patients suffering from measles were screened for malnutrition and those found malnourished were referred to the nutrition rehabilitation units of county referral hospitals for appropriate care using the WHO protocol on Integrated Management of Childhood Illnesses (IMCI) [16].

**Immunization:** routine immunization coverage has been improving in the aftermath of the Ebola virus disease outbreak. In 2014, routine immunization coverage dropped to the lowest in a decade due to the EVD outbreak which disrupted the provision of routine health services across the country. However, in 2016 and 2017 the country was able to achieve the target of 80% or above measles immunization administrative coverage. Despite the national average at 80% and 87% in 2016 and 2017 respectively, Montserrado and Nimba counties, the most two populous counties fell below the 80% mark. Additional analysis of counties that achieved the 80% at district level, 10% of the 92 health districts had not achieved 80% coverage or above in the last 5 years. Circumscribed reactive measles immunization campaigns were conducted in the districts with outbreaks coordinated by the district and county rapid response teams (CRRT) and (DRRT) as a response measure to measles outbreaks on addition to intensification of routine immunization in affected counties, however vaccine hesitancy continues to affect the coverage especially in the Mandingo and Fula tribes’ communities. The local epidemiology of the disease informed the target age group for each reactive measles campaign.

**Social mobilization and community engagement:** during measles outbreaks in Liberia, community engagement and awareness activities are intensified in affected communities aimed at encouraging the population to utilize routine vaccination services, reactive measles campaigns reporting all suspected cases of measles to health facilities early. Traditional healers are also engaged to refer all suspected measles patients to the nearest health facilities. Community health workers, volunteers, leaders of places of worship, markets leaders and women groups were used for risk communication guided by ministry of health and county health promotion units. Messages to the community were brief, concise and translated in local dialects which were transmitted through local radios, jingles, posters and fliers. The messages provided information about the disease with focus on signs and symptoms, prevention through measles vaccination and encouragement to seek care at the nearby health-care facility early after symptom onset. Community engagement meetings were held with community, religious and political leaders, and presentations at markets, health centers, town hall meetings, schools and places of worship.

Lessons learnt: a key lesson in this context is the fact that low immunization coverage is a recipe for outbreaks. The use of community informants for detection and reporting priority diseases of which measles is among increase measles alerts reporting hence contributing to early measles outbreak detection. Community engagement is a critical part of response and control efforts. Involvement of communities through their chiefs, elders, women and youth groups builds trust and facilitates cooperation from community members during response activities.

**Limitations:** few children had vaccination cards to verify routine vaccination history. However, even among children with cards, measles coverage remained low (78%) for all counties combined (data not shown). Majority of the measles cases reported (55%) vaccination status was unknown; this posed a challenge in data analysis and interpretation of study findings. It was difficult to establish whether the large number of cases with unknown vaccination was non-vaccinated or vaccinated due to missing records, incomplete data and recall challenge from the caretakers. It can also be partly attributed to the poor quality of information asked for recall of vaccination status by health workers. Information about reactive campaigns following measles outbreak was only available in 4 out of 10 counties (40%).

**Recommendations:** given that measles incidence is particularly very high among age group 1 to 10 years, which constitute 65% of all confirmed cases, need to conduct a national campaign targeting 6 months to 15 years. Liberia still offers one dose of measles vaccine in the routine immunization schedule. There is need to introduce the second dose measles vaccine in the routine immunization schedule of the country. Strengthening of surveillance and epidemic preparedness and response with emphasis on ensuring emergency resources are readily available at sub-national level for quick response need to be prioritized. Strengthen community engagement to ensure a higher uptake of immunization services.

## Conclusion

Revitalization of IDSR as a lesson learnt from Ebola outbreak has provided immense opportunity for improving case detection and response to measles. The introduction of booster doses as part of routine immunization need to be considered. Supplemental immunization campaign and strengthening of routine immunization core activities need prioritization as a hall mark to reduce the persistence of measles and prevent out breaks in Liberia, West Africa and AFRO region.

**Availability of data and materials:** the identified data used and/or analysed during this documentation is available from the corresponding authors and a property of Liberia ministry of health.

### What is known about this topic

- Measles is a highly contagious respiratory disease;
- Measles typically starts with fever, runny nose, cough, red eyes and sore throat followed by Koplik spots, or tiny white spots developing inside the mouth and a rash that starts on the face spreading downward to the rest of the body;
- Measles can be prevented with the MMR (measles, mumps, rubella) vaccine, which is safe and effective.

### What this study adds

- Accumulation of a large susceptible population with an immunity gap is a risk for large scale measles outbreaks as seen in Liberia where 51% of measles cases were above 5 years;
- Circumscribed reactional measles vaccination is good at interrupting transmission of measles during outbreaks hence reducing morbidity due to measles;
- Measles vaccination in life time is protection for measles infection.

## Competing interests

The authors declare no competing interest.
